# Mechanisms by Which Interleukin-12 Corrects Defective NK Cell Anticryptococcal Activity in HIV-Infected Patients

**DOI:** 10.1128/mBio.00878-16

**Published:** 2016-08-23

**Authors:** Stephen K. Kyei, Henry Ogbomo, ShuShun Li, Martina Timm-McCann, Richard F. Xiang, Shaunna M. Huston, Anutosh Ganguly, Pina Colarusso, M. John Gill, Christopher H. Mody

**Affiliations:** a The Calvin, Phoebe and Joan Snyder Institute for Chronic Diseases, University of Calgary, Calgary, Alberta, Canada; b Department of Microbiology, Immunology and Infectious Diseases, University of Calgary, Calgary, Alberta, Canada; c Department of Pathology and Laboratory Medicine, University of Calgary, Calgary, Alberta, Canada; d Department of Internal Medicine, University of Calgary, Calgary, Alberta, Canada; e Department of Physiology and Pharmacology, University of Calgary, Calgary, Alberta, Canada

## Abstract

*Cryptococcus neoformans* is a pathogenic yeast and a leading cause of life-threatening meningitis in AIDS patients. Natural killer (NK) cells are important immune effector cells that directly recognize and kill *C. neoformans* via a perforin-dependent cytotoxic mechanism. We previously showed that NK cells from HIV-infected patients have aberrant anticryptococcal killing and that interleukin-12 (IL-12) restores the activity at least partially through restoration of NKp30. However, the mechanisms causing this defect or how IL-12 restores the function was unknown. By examining the sequential steps in NK cell killing of *Cryptococcus*, we found that NK cells from HIV-infected patients had defective binding of NK cells to *C. neoformans*. Moreover, those NK cells that bound to *C. neoformans* failed to polarize perforin-containing granules to the microbial synapse compared to healthy controls, suggesting that binding was insufficient to restore a defect in perforin polarization. We also identified lower expression of intracellular perforin and defective perforin release from NK cells of HIV-infected patients in response to *C. neoformans*. Importantly, treatment of NK cells from HIV-infected patients with IL-12 reversed the multiple defects in binding, granule polarization, perforin content, and perforin release and restored anticryptococcal activity. Thus, there are multiple defects in the cytolytic machinery of NK cells from HIV-infected patients, which cumulatively result in defective NK cell anticryptococcal activity, and each of these defects can be reversed with IL-12.

## IMPORTANCE

The mechanisms by which NK cells bind directly to pathogens and deploy their deadly cytolytic machinery during microbial host defense are only beginning to be elucidated. With the goal of understanding this process, we used NK cells from HIV-infected patients, which were known to have a defect in killing of *Cryptococcus neoformans*. Taking advantage of previous studies that had shown that IL-12 restored killing, we used the cytokine as a gain-of-function approach to define the relevance of multiple steps in the recognition and cytolytic pathway. We demonstrated that NK cells from HIV-infected patients failed to kill *Cryptococcus* due to defects in perforin expression, granule polarization, and release of perforin. Additionally, IL-12 restored recognition of *C. neoformans* through binding of the NK-activating receptor NKp30. These observations identify important mechanisms used by NK cells to kill microbes and determine that defects in NK cells from HIV-infected patients are reversible.

## INTRODUCTION

Natural killer (NK) cells are more than just innate immune lymphocytes that are critical in cytolytic defense against tumor and virus-infected cells (1, 2). Indeed, the antimicrobial activity of NK cells extends to bacteria and parasites by direct recognition and triggering their cytolytic function (3). Depletion of murine NK cells *in vitro* has been shown to compromise immune defense against various bacteria and parasites, including *Pseudomonas aeruginosa* (4), *Citrobacter rodentium* (5), trypanosomes (6), and mycobacteria (7). In cryptococcosis, NK cells and not polymorphonuclear cells (PMN) in the beige mouse model are responsible for killing (8). Additionally, cellular depletion *in vivo* impaired clearance of *Cryptococcus neoformans*, which was restored by transfer of NK cells to the depleted mice (9), and antibody depletion of NK cells caused an increase in the burden of *C. neoformans* in the lung after an intravenous inoculation of the organisms (10). Moreover, murine and human NK cells have direct antimicrobial activity against a variety of fungi, including *Cryptococcus neoformans*, *Aspergillus fumigatus*, *Candida albicans*, *Paracoccidioides brasiliensis*, and *Rhizopus oryzae* (11–16).

The mechanisms of NK cell cytotoxicity have been studied extensively for tumor killing, but very little is known about direct NK cell cytotoxicity for microbes. During tumor killing, the process involves a series of steps, initiated by binding of activating NK receptors to their ligands (17), stimulating complex intracellular signals (18), leading to actin polymerization, microtubule reorganization, and convergence of the secretory granules to the microtubule organizing center (MTOC) (19). The MTOC together with lytic granules is then polarized to the immunological synapse, where the lytic granules dock and fuse with the plasma membrane, leading to the extracellular release of granule contents that ultimately kill the target cell (19, 20). The major cytolytic granule proteins include perforin and granulysin (membrane-disrupting proteins) and granzymes (serine proteases) (21). However, it is not clear whether these sequential processes are involved in NK cell-mediated microbial killing.

The effector molecule perforin plays an essential role in NK cell antimicrobial activity. Perforin is required for the control of intracellular bacterial infections, such as those with *Mycobacterium tuberculosis* (7), and is used by NK cells for antifungal cytotoxicity against fungi such as *A. fumigatus*, *Rhizopus oryzae*, and *C. neoformans* (15, 16, 22). However, it is not known whether perforin polarization is required for fungal killing by NK cells. Thus, defects in binding, intracellular signal transduction, lytic granule transport, polarization, and release, as well as effector molecule production and activation, could lead to impaired direct NK cell-mediated antimicrobial activity.

Cryptococcal meningitis is the most common fungal infection of the central nervous system in AIDS patients (23). In high-HIV-prevalence regions such as those in sub-Saharan Africa, *Cryptococcus neoformans* is the leading cause of meningitis and is more common than *Neisseria meningitidis* and *Streptococcus pneumoniae* (24). Despite the availability of antiretroviral therapy (ART), 1 million cases of cryptococcal meningitis occur annually worldwide, with a mortality of 68% within the first 3 months of diagnosis (25). It is also notable that some 8.4% of HIV-infected asymptomatic patients have high levels of cryptococcal antigenemia regardless of CD4 count, suggesting that these patients have a permissive immune defect and subclinical infection (26). Therefore, it is relevant and important to determine defects that might predispose patients to a permissive immune defect and subclinical infection leading to continual antigenemia with the goal of developing immunologic approaches to clear the pathogen.

In view of the importance of NK cells for cryptococcal host defense, it is particularly concerning that NK cells from HIV-infected patients have impaired function (27). These NK cells have various phenotypic and functional defects, such as high expression of inhibitory natural killer receptors (iNKRs), low levels of natural cytotoxicity receptors, and reduced cytotoxic capacity for tumor cells (27–29). ART restores many aspects of immunity in HIV-infected patients (30, 31), including some aspects of NK cell immunity, such as restoring expression of 2B4 and reducing inhibitory NK receptor activity (27, 32). However, certain critical functions of NK cells remain compromised, including low levels of NK cell-activating receptors (27). We therefore sought to investigate whether the cytolytic machinery of NK cells in response to *C. neoformans* was compromised in HIV-infected patients receiving ART.

A study performed prior to widespread use of ART showed that NK cells from HIV-infected patients had defective anticryptococcal activity and that *ex vivo* treatment with interleukin-12 (IL-12) restored activity (33). However, it was unclear whether this defect is still present and relevant in the current era of treatment with suppression of viral replication and immune reconstitution, and if it does persist, what mechanisms underlie this defective anticryptococcal activity, or how IL-12 restores these defects. We therefore used IL-12 as a tool to decipher the defects in the cytolytic machinery in NK cells from HIV-infected patients and to restore NK cell functions. NK cell functions were systematically studied, including surface receptor expression and binding of *C. neoformans* to NK cells as assessed by flow cytometry, perforin polarization as assessed by immunofluorescent microscopy, perforin content within cells as assessed using flow cytometry, and perforin release as assessed using enzyme-linked immunosorbent assay (ELISA).

## RESULTS

### IL-12 restored antifungal activity of NK cells from HIV-infected patients via a perforin-dependent mechanism.

**FIG 1:**
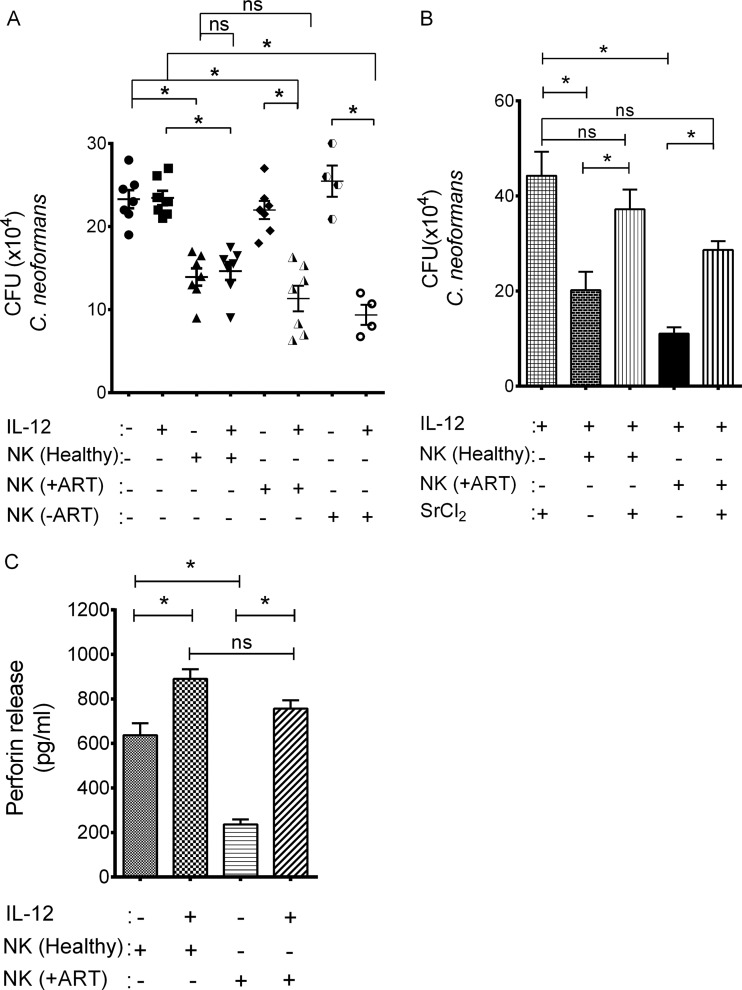
IL-12 restored defective granule-mediated antifungal activity of NK cells from HIV-infected patients. (A) NK cells from healthy donors, HIV-infected patients on ART (+ART), and HIV-infected patients with no ART (−ART) were isolated and pretreated with rh-IL-12 for 20 h or not pretreated, and antifungal activity was assessed against *C. neoformans*. Data shown are from *n* = 7 independent healthy controls and 7 independent HIV-infected patients receiving ART and *n* = 4 independent patients not receiving ART. (B) NK cells from healthy or HIV-infected patients on ART were pretreated with rh-IL-12 for 18 h and then incubated in the presence or absence of SrCl_2_ for 24 h. Treated NK cells were incubated with *C. neoformans*, and antifungal activity was determined by counting CFU. Data shown are the mean from *n* = 5 independent healthy controls and *n* = 3 independent HIV-infected subjects. (C) NK cells from healthy donors and HIV-infected patients on ART were pretreated with rh-IL-12 for 18 h. The stimulated perforin release in response to *C. neoformans* was assessed by ELISA. Data shown are the mean from *n* = 4 independent healthy controls and *n* = 4 independent HIV-infected patients. Data are the mean ± standard error of the mean. *, *P* < 0.05; ns, not significant.

It had previously been shown that NK cells from ART-naive HIV-infected patients had defective anticryptococcal activity (33). We were interested in determining whether the impaired anticryptococcal activity was similar in HIV-infected patients who were receiving ART and whether IL-12 restored the activity in both groups of patients. We therefore cocultured freshly isolated NK cells from ART-receiving and ART-naive HIV-infected patients with *C. neoformans* and assessed anticryptococcal activity. NK cells from healthy donors had significant anticryptococcal activity, while NK cells from either ART-naive patients or patients receiving ART did not (Fig. 1A). To determine if IL-12 restored this defect, we treated NK cells from healthy donors, HIV-infected patients receiving ART, and ART-naive HIV-infected patients with recombinant human IL-12 (rh-IL-12) and assessed cryptococcal CFU. NK cells from healthy donors showed no increase in anticryptococcal activity following IL-12 treatment (Fig. 1A). In contrast, NK cells from patients receiving ART and from patients who were ART naive showed a significant increase in their anticryptococcal activity that was not significantly different from the level of killing by untreated NK cells from healthy subjects (Fig. 1A). Thus, NK cells from both HIV patients receiving ART and ART-naïve HIV patients had defective anticryptococcal activity that could be reversed with IL-12.

NK cells kill *Cryptococcus* by degranulation of secretory lysosomes that contain the effector molecule perforin (34). Consequently, we asked whether IL-12 restored anticryptococcal activity via degranulation rather than employing some other non-granule-mediated mechanism. Because we were unable to use small interfering RNA (siRNA) knockdown in primary NK cells from HIV-infected patients, we depleted granules from NK cells using SrCl_2_ as previously described (22). SrCl_2_ treatment reduced the anticryptococcal activity of IL-12-treated NK cells from both HIV-infected patients receiving ART and healthy donors (Fig. 1B), similarly to previous studies using NK cells from healthy subjects (22), suggesting that the enhanced killing was granule mediated. To determine whether the defect in NK cells from HIV-infected patients receiving ART was associated with defective release of perforin, NK cells from both healthy and HIV patients were challenged with *C. neoformans* and the stimulated perforin released was assessed by ELISA. Untreated NK cells from HIV-infected patients showed an impaired ability to release perforin after *C. neoformans* exposure compared to healthy donors (Fig. 1C), consistent with our previous observation (35). To investigate whether IL-12 enhanced NK cell perforin release from HIV-infected patients, we treated NK cells from healthy donors and HIV-infected patients with recombinant human IL-12 and assessed their ability to secrete perforin in response to *C. neoformans* stimulation. IL-12-treated NK cells from HIV-infected patients showed an approximately 300% increase in perforin release in response to *C. neoformans* compared to NK cells from HIV-infected patients not treated with IL-12, which was above the threshold of release needed for killing by NK cells from healthy subjects (Fig. 1C). In contrast, there was only a 60% increase in perforin release in NK cells from healthy subjects (Fig. 1C), which failed to result in further increased killing by NK cells from healthy subjects, suggesting that a threshold of release was more important than an incremental increase. Thus, NK cells from ART-receiving HIV-infected patients had a defect in cryptococcal killing that was restored with IL-12, via perforin degranulation.

### NK cells from HIV-infected patients had lower perforin expression that was restored by IL-12 treatment.

**FIG 2:**
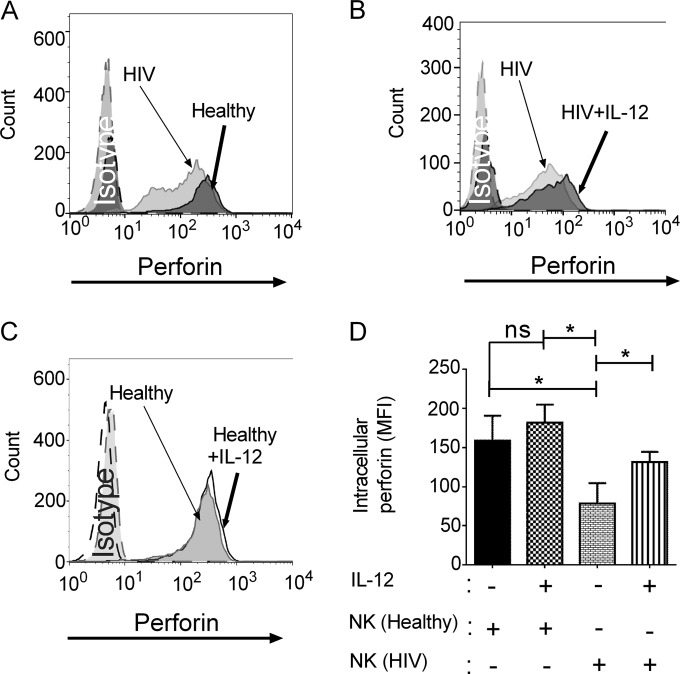
NK cells from HIV-infected patients had lower perforin expression that was restored by IL-12 treatment. (A) NK cells from healthy donors and HIV-infected patients on ART were freshly isolated, made permeable, labeled with antiperforin antibody, and analyzed by flow cytometry. (B) NK cells from HIV-infected patients on ART were freshly isolated, treated with IL-12 or not treated, made permeable, labeled with antiperforin antibody, and analyzed by flow cytometry. (C) NK cells from healthy donors were freshly isolated, treated with IL-12 or not treated, made permeable, labeled with antiperforin antibody, and analyzed by flow cytometry. Data in panels A to C are from one of 5 representative experiments. (D) Mean fluorescence intensity of perforin labeling in IL-12-treated NK cells. Data shown are the mean from *n* = 5 independent healthy controls and *n* = 5 independent HIV-infected patients. Data are the mean ± standard error of the mean. *, *P* < 0.05; ns, not significant.

We considered the possibility that NK cells from HIV-infected patients might have defective perforin stores and that defective stores might be an additional mechanism underlying the defective killing. To address this possibility, freshly isolated NK cells were made permeable and labeled with fluorescein isothiocyanate (FITC)-conjugated antiperforin antibody that recognizes the active form of perforin responsible for NK cell cytotoxic functions. NK cells from patients receiving ART showed a lower level of fluorescence as determined by flow cytometry than did NK cells from healthy donors, suggesting that perforin content was lower (Fig. 2A). We then asked whether increased perforin expression could provide a possible explanation for increased perforin release from NK cells of HIV-infected patients in response to IL-12 treatment. To investigate whether IL-12 enhanced the intracellular expression of perforin in NK cells from HIV-infected patients, we treated NK cells from healthy donors and HIV-infected patients with recombinant human IL-12 and assessed intracellular perforin content by flow cytometry. In response to IL-12, there was an increased level of fluorescence detected with the anti-perforin antibody in NK cells from HIV-infected patients (Fig. 2B), compared with NK cells from healthy donors (Fig. 2C). While there was only a modest and not significant increase in mean fluorescent intensity after IL-12 treatment of NK cells from healthy subjects, IL-12-treated NK cells from HIV-infected patients expressed a higher level of fluorescence (Fig. 2D). These studies are consistent with a process whereby the perforin content in untreated NK cells from HIV-infected patients was low and increased above the threshold required to kill in response to IL-12. In contrast, the modest increase in perforin content in NK cells from healthy subjects failed to increase killing, suggesting that a threshold of release was more important than an increase above that threshold. Together, these findings provide an explanation for the enhanced perforin release and anticryptococcal activity (Fig. 1).

### NKp30-dependent binding of *C. neoformans* to NK cells from HIV-infected patients is restored with IL-12.

**FIG 3:**
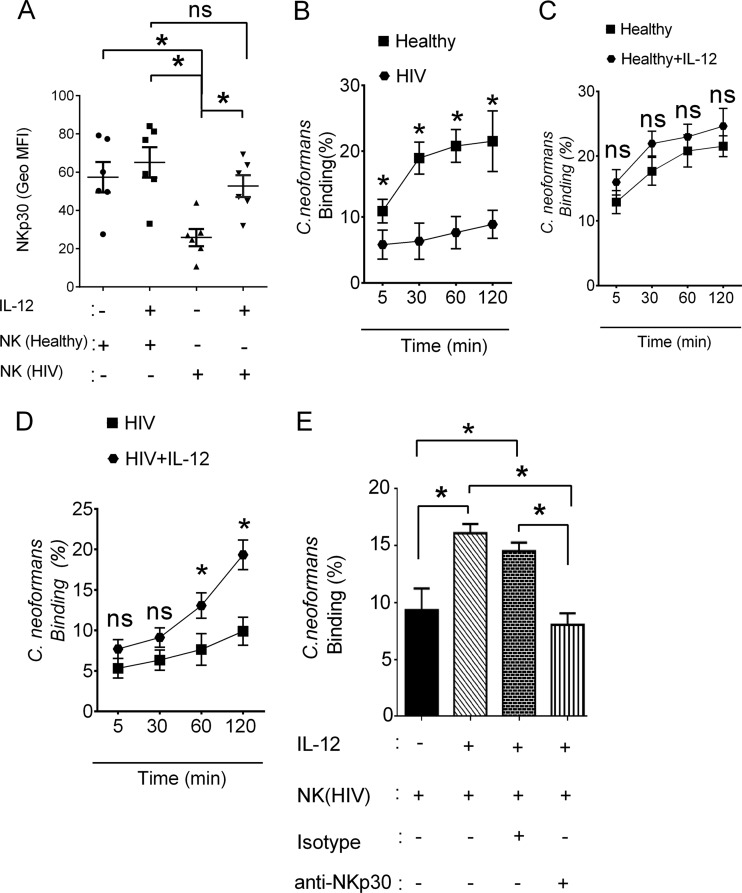
IL-12 restored NKp30-dependent binding of *C. neoformans* to NK cells from HIV-infected patients. (A) NK cells from HIV-infected patients on ART were freshly isolated, treated with IL-12 for 24 h or not treated, labeled with anti-NKp30 antibody, and analyzed by flow cytometry. Data shown are the mean from *n* = 6 independent healthy donors and *n* = 6 independent HIV-infected patients. Statistical significance was determined using an unpaired *t* test. *, *P* < 0.05, for all panels; ns, not significant. (B) CD11a-PE-Cy5-labeled NK cells from healthy donors and HIV-infected patients were cocultured with FITC-labeled *C. neoformans* for different lengths of time. Conjugates were detected when both red (NK cells) and green (*Cryptococcus*) fluorescence were detected in the same event by flow cytometry. Data shown are the mean from *n* = 4 independent healthy controls and *n* = 4 independent HIV-infected subjects. (C) NK cells from healthy donors were treated with IL-12 for 18 h or not treated and were cocultured with *C. neoformans* for different lengths of time. Conjugates were detected as described above. Data shown are the mean from *n* = 4 independent healthy controls treated with or without IL-12. (D) NK cells from HIV-infected subjects were treated with IL-12 for 18 h or not treated and were cocultured with *C. neoformans* for different times. Data shown are the mean from *n* = 4 independent HIV-infected subjects treated with IL-12 or not treated. (E) NK cells from HIV-infected subjects were treated with IL-12 for 18 h or not treated. The cells were preincubated with polyclonal anti-NKp30 isotype control for 45 min or without antibody, before assessment of conjugate formation with *C. neoformans* at 1 h. Data shown are the mean from *n* = 3 independent HIV-infected patients. Data are the mean ± standard error of the mean.

Having previously demonstrated that defective anticryptococcal activity was associated with defective NKp30 expression (35), we were interested in determining whether NKp30 expression conferred greater binding. Consistent with our previous observation, NK cells from HIV-infected patients had lower surface expression of NKp30 than did those from healthy subjects; however, *ex vivo* IL-12 treatment significantly increased NK cell surface expression of NKp30 in HIV-infected subjects (Fig. 3A). To assess whether NK cells from HIV-infected patients were able to bind to *C. neoformans*, we labeled the NK cells with phycoerythrin (PE)-Cy5-conjugated anti-CD11a and then cocultured them with FITC-labeled *C. neoformans* over time. It is worth noting that direct NK cell anticryptococcal activity is preceded by LFA-1 (CD11a/CD18)-independent binding of NK cells (36). Conjugates were identified when red fluorescence (NK cell) and green fluorescence (*C. neoformans*) were detected in the same event by flow cytometry. The percentage of *C. neoformans* cells that formed conjugates with NK cells from HIV-infected patients receiving ART was lower than those for NK cells from healthy control subjects (Fig. 3B). FITC labeling did not affect cryptococcal activity (see Fig. S1A in the supplemental material) or binding (see Fig. S1B). To investigate whether IL-12 also restored binding of NK cells from HIV-infected patients to *C. neoformans*, NK cells were treated with IL-12, or not treated, and conjugates with *C. neoformans* were assessed. IL-12-treated NK cells from healthy subjects did not show a significant increase in the percentage of *Cryptococcus* organisms that formed conjugates (Fig. 3C). In contrast, IL-12-treated NK cells from HIV-infected patients showed a significant increase in conjugate formation (Fig. 3D). Together, these observations suggested that IL-12 influences the expression of the receptor required for NK cell binding to *Cryptococcus* in HIV-infected patients.

To determine whether NKp30 is the receptor that confers increased binding of IL-12-treated NK cells from HIV-infected patients to *C. neoformans*, IL-12-treated NK cells from HIV-infected patients were incubated with anti-NKp30 blocking antibody or isotype-matched control antibody, prior to assessing their ability to bind *C. neoformans*. Blocking NKp30 significantly reduced the percentage of conjugates formed by IL-12-treated NK cells from HIV-infected patients (Fig. 3E), which correlated with a 60% reduction in killing (35). These observations provided evidence that reduced binding of NK cells from HIV-infected patients to *C. neoformans* was dependent on NKp30 expression, which was restored by IL-12 treatment.

### NK cells from HIV-infected patients failed to polarize perforin in response to *C. neoformans.*

**FIG 4:**
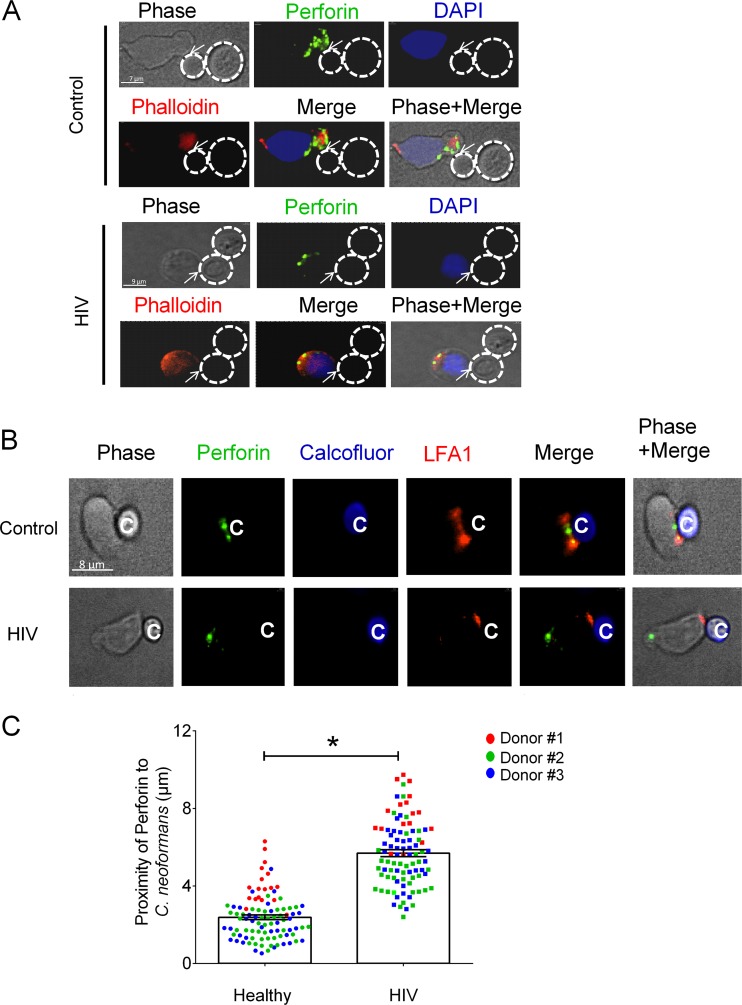
NK cells from HIV-infected patients failed to polarize perforin to the area of contact with *C. neoformans*. NK cells from healthy donors and patients receiving ART were challenged with *C. neoformans* for 1 h. (A) Location of perforin in NK cells from healthy controls and HIV-infected subjects bound to *C. neoformans*. The NK cells were labeled with antiperforin antibody (green), DAPI for nucleus (blue), and phalloidin for F-actin (red). The traced outline corresponds to the perimeter of *C. neoformans* from the phase-contrast image and is overlaid on the fluorescence image in all panels. (B) NK cells from healthy and HIV-infected patients were challenged with *C. neoformans* for 1 h. The NK cells were labeled with antiperforin antibody (green) and anti-LFA-1 (red). *C. neoformans* cells were labeled with calcofluor (blue). (C) The distances of perforin to *C. neoformans* in NK cells from 3 healthy controls and 3 HIV-infected patients in 3 experiments were determined with Volocity software. Each data point is the mean distance of 15 to 30 granules of 18 (experiment 1; red circles), 44 (experiment 2; green circles), and 35 (experiment 3; blue circles) control NK cells and 17 (experiment 1; red squares), 40 (experiment 2; green squares), and 36 (experiment 3; blue squares) HIV-infected patient NK cells that had formed conjugates with *C. neoformans*. Data are the mean ± standard error of the mean. *, *P* < 0.05.

NK cells kill tumor cells by mobilizing, polarizing, and deploying their cytolytic granules (17). Having demonstrated that fewer NK cells from HIV-infected patients bind to *C. neoformans*, we investigated whether this caused defective granule mobilization and polarization by NK cells that were able to bind *C. neoformans*. If mobilization and polarization were impaired, perforin molecules would fail to localize at the microbial synapse as a prerequisite for release. NK cells from healthy donors and patients receiving ART were cocultured with *C. neoformans* over time, fixed, made permeable, labeled with FITC-conjugated antiperforin antibody, and assessed for proximity of the perforin-containing granules to sites of fungal attachment. The distance of perforin to the point of contact with *C. neoformans* was used to reflect the extent to which perforin was polarized to the microbial synapse. Images demonstrated that perforin-containing granules were farther from the area of contact with *C. neoformans* in NK cells from HIV-infected patients than in NK cells from healthy donors (Fig. 4A). The increased distance of perforin to the point of contact with *Cryptococcus* suggested that the process leading to polarization was impaired in HIV-infected patients. This defect in polarization provided a mechanism by which NK cells from HIV-infected patients receiving ART had defective killing. However, we considered the possibility that NK cells from HIV-infected patients failed to polarize perforin because they made contact but did not form a synapse with *C. neoformans*. To address this possibility, we examined LFA-1, which had been shown to accumulate at the NK cell-cryptococcal synapse (36). Images demonstrated that LFA-1 accumulates at the NK cell-cryptococcal synapse, in NK cells from both HIV-infected patients (Fig. 4B, bottom) and healthy donors (Fig. 4B, top) despite the observation that perforin-containing granules were more remote from the area of contact with *Cryptococcus* in NK cells from HIV-infected patients (Fig. 4B). Quantitative analysis of proximity of perforin to *Cryptococcus* confirmed the observations in the images (Fig. 4C). Similarly, three-dimensional (3D) image analysis confirmed the appearance of a synapse between NK cells and *C. neoformans* (see Movies S1 and S2 in the supplemental material).

### IL-12 restored perforin polarization in NK cells from HIV-infected patients in response to *C. neoformans.*

**FIG 5:**
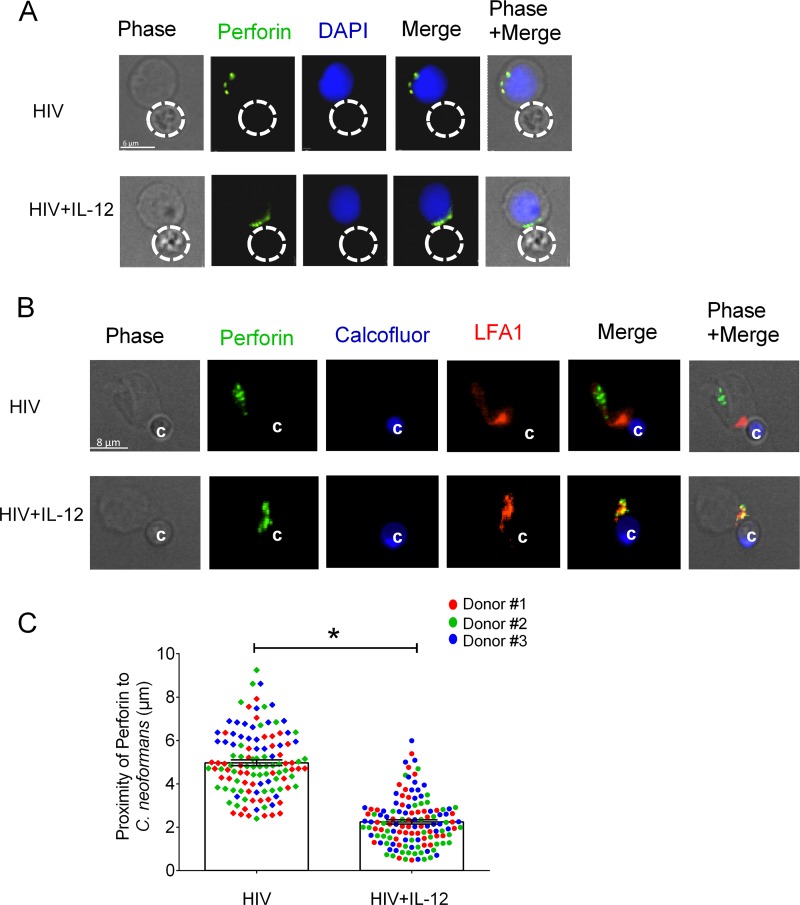
IL-12 restored polarization of perforin in NK cells from HIV-infected patients. NK cells from HIV-infected patients were treated with IL-12 for 20 h or not treated and were challenged with *C. neoformans* at an effector-to-target ratio of 1:2 for 1 h. (A) Localization of perforin in non-IL-12-treated and IL-12-treated NK cells from HIV-infected subjects bound to *C. neoformans*. The NK cells were labeled with antiperforin antibody (green) and DAPI for the nucleus (blue). The traced outline corresponds to the perimeter of *C. neoformans* from the phase-contrast image and is overlaid on the fluorescence image in all panels. (B) NK cells from HIV-infected patients were treated with IL-12 for 20 h or not treated and were challenged with *C. neoformans* at an effector-to-target ratio of 1:2 for 1 h. The NK cells were labeled with antiperforin antibody (green) and anti-LFA-1 (red). *C. neoformans* was labeled with calcofluor (blue). (C) The distances of perforin to *C. neoformans* in NK cells from HIV-infected subjects (HIV) and IL-12-treated NK cells from the same HIV-infected patients (HIV+IL-12) in 3 separate experiments were determined with Volocity software. Each data point is the mean distance of 15 to 30 granules of 44 (experiment 1; red squares), 46 (experiment 2; green squares), and 30 (experiment 3; blue squares) NK cells of an HIV-infected patient and 43 (experiment 1; red circles), 46 (experiment 2; green circles), and 46 (experiment 3; blue circles) IL-12-treated NK cells of an HIV-infected patient that had formed conjugates with *C. neoformans*. Data are the mean ± standard error of the mean. *, *P* < 0.05.

To investigate whether IL-12 restored defective perforin polarization in NK cells from HIV-infected patients, we treated NK cells from patients receiving ART with recombinant human IL-12 prior to challenge with *Cryptococcus*. The images demonstrated that perforin-containing granules were in closer proximity to the area of NK cell contact with *Cryptococcus* in IL-12-treated NK cells from HIV-infected patients (Fig. 5A, bottom) than in untreated NK cells from HIV-infected patients (Fig. 5A, top). As expected, IL-12-treated NK cells from HIV-infected patients recognized *Cryptococcus* as evidenced by LFA-1 accumulation at the NK cell-cryptococcal synapse (Fig. 5B). Quantitative image analysis showed that perforin-containing granules were closer to the point of NK cell contact with *C. neoformans* in IL-12-treated NK cells than in untreated NK cells from HIV-infected patients (Fig. 5C). These observations provided an additional mechanism by which IL-12 restored killing by NK cells from HIV-infected patients. However, more importantly, they demonstrate, using loss- and gain-of-function approaches, that polarization is required for NK cell killing of *Cryptococcus*. These observations suggest that defective perforin expression in NK cells from HIV-infected patients, in addition to defective polarization, contributed to the defective NK cell killing of *Cryptococcus* and that these defects were corrected with IL-12.

## DISCUSSION

In this study, we made 5 key observations about defective fungal killing by NK cells from HIV-infected patients. (i) Defective direct anticryptococcal killing by NK cells from ART-naive patients was not restored in patients receiving ART. (ii) These cells also demonstrated low levels of perforin expression and defective perforin release. (iii) These NK cells had defective binding in response to *C. neoformans* that was at least partially due to reduced NKp30 expression. (iv) NK cells from patients receiving ART had defective perforin-containing granule polarization to the fungal target despite evidence of binding and LFA-1 accumulation at the synapse. (v) IL-12 treatment restored these defects.

Several studies have shown phenotypic and functional defects in NK cells from HIV-infected patients, including aberrant antibody-dependent cellular cytotoxicity; high expression of inhibitory natural killer receptors; low levels of activating receptors, including NKp30, NKp44, and NKp46 (37); and reduced tumor cytotoxicity (27, 29, 38, 39). It has also been reported that defective NK cells in HIV-infected patients are a result of a shift in NK cell subsets with reduced numbers of the more cytotoxic form and increased numbers of the less cytotoxic subset (40). Other theories propose that excessive activation of NK cells leads to functional defects as a result of exhaustion (41). Our observations contribute to this body of literature by demonstrating functional defects in NK cells that can be reversed by IL-12. These defects were unlikely to be due to a change in differentiation or exhaustion since we demonstrated reversibility, and IL-12 tends to exacerbate exhaustion in T cells (42). Only a few studies have investigated binding or granule trafficking as a possible explanation for defective cytotoxicity by NK cells from HIV-infected patients. Studies using tumor cells as targets demonstrated that NK cells from HIV-infected patients had decreased ability to form conjugates with K-562 and U-937 tumor cell lines (43, 44). It has been reported that NK cells from HIV-infected patients have a defective ability to rearrange tubulin after forming conjugates with tumor cells (43), which consequently leads to aberrant cytolytic capacity. Our results suggest that the aberrant NK cytotoxicity of HIV-infected patients is a result of a defect in granule mobilization and deployment to microbial target cells.

Many studies have implicated the cytolytic machinery in NK cell-mediated antifungal activity. Previous studies have demonstrated that NK cells form conjugates with *C. neoformans* and that contact is required for anticryptococcal activity (13, 22, 45). Other studies have demonstrated that killing of several fungal species by NK cells is cytolytic granule dependent (3, 11, 22). Knockdown of perforin but not granulysin by small interfering RNA abrogated NK cell anticryptococcal activity (22). Further, perforin-mediated antifungal activity is not exclusive to *C. neoformans* but occurs against multiple fungal pathogens, including *A. fumigatus* and *Rhizopus oryzae* (15, 16). Enriched human NK cells lost their anticryptococcal activity after treatment with monensin, a known inhibitor of Golgi transport (13), which is consistent with a requirement for granule loading during NK anticryptococcal activity. Importantly, it has been demonstrated that the contents of cytolytic granules are required by NK cells to kill *C. neoformans* (46). Our results provide the evidence that granule trafficking and polarization of perforin to the microbial synapse are required for NK cell-mediated anticryptococcal activity, since NK cells from HIV-infected patients do not polarize and do not kill, while NK cells from healthy subjects or IL-12-treated NK cells from HIV-infected patients polarize their lytic granules and kill the pathogen.

We identified multiple defects that may have implications for other NK cell responses, such as NK cell antitumor activity and antiviral responses (47, 48). Using IL-12 to dissect these defects, we showed that NK cells from HIV-infected patients have defective binding to the target cell that is dependent on the expression of NKp30. Additionally, we demonstrated that NK cells from HIV-infected patients had defective polarization of perforin to their target and released a smaller amount of perforin. While binding is a prerequisite for signal transduction leading to polarization, we cannot exclude the possibility that binding and perforin polarization are independent defects since residual NK cells from HIV-infected patients that bound to *C. neoformans* failed to polarize perforin to the target. Further, we found that restoring intracellular perforin content in NK cells from HIV-infected patients was associated with restoration of killing. These observations suggest that there are multiple defects in the cytolytic machinery of NK cells from HIV patients.

Our observation that IL-12 restored the defective anticryptococcal activity of NK cells from HIV-infected patients highlights the importance of IL-12 in host NK cell immune responses. Importantly, HIV-infected patients have aberrant IL-12 production (49). The mechanism involves HIV-vpr-mediated suppression of IL-12 p35 production, resulting in defective release of the biologically active IL-12 heterodimer by monocytic cells (49). The observation that IL-12 modestly increased perforin expression and release in NK cells from healthy subjects with no additional increase in killing of the fungi suggests that a threshold of perforin release is required for this process. Unlike the situation in healthy subjects, IL-12 likely potentiates antifungal activity of NK cells from HIV-infected subjects by compensating for defects in the NK cell perforin-mediated killing pathway. Supporting this model is the observation that IL-12 mediates NK cell antitumor effects via the perforin pathway (50, 51) and increased expression of activating receptors such as NKp46 (52). IL-12 increases perforin in NK cells with a consequent increase in NK cell cytotoxicity (51, 53). Further, IL-12 has been shown to enhance NK cell release of granule-derived proteins, including serine proteases (54), suggesting that IL-12 potentiates NK cell cytotoxicity rather than initiating the process.

Several studies have demonstrated the importance of IL-12 in the treatment of opportunistic infections as well as tumors (55, 56). Despite its toxicity in patients, IL-12 therapy has been effectively administered to patients with Kaposi’s sarcoma, when other therapies have failed (57, 58). New therapeutic approaches have demonstrated successful use of IL-12 in treating leukemia (59, 60), suggesting that IL-12 therapy may be beneficial. IL-12 that was administered in the absence of antiretroviral therapy to Indian rhesus macaques infected with simian immunodeficiency virus led to partial restoration of NK lytic functions (61). In fact, IL-12 has been demonstrated to be a potent cytokine in the host antifungal immune response. IL-12 synergizes with antifungal agents such as fluconazole to reduce the number of yeast cells in the brain, lung, and liver, as well as in conjunction with amphotericin B against *Histoplasma capsulatum* infection in mice (62, 63). Further, IL-12 can protect BALB/c mice susceptible to *Coccidioides immitis* infection by switching the immune response from a nonprotective Th2 to a protective Th1 response (56). Importantly, IL-12 has been shown to be essential in NK cell anticryptococcal activity. Mice with a targeted deletion of IL-12, infected with *Cryptococcus neoformans*, had higher numbers of the fungi in both brain and lung than did controls (64).

Because of its efficacy, it is tempting to consider IL-12 for therapy. However, systemic toxicity may limit its therapeutic use. Possibly, combinations of cytokines that would have the same effect might be considered. That combination of cytokines could be selected on their ability to stimulate Jak kinases (Tyk-2 and Jak-2), leading to signaling through STAT-4, which is the canonical pathway triggered by the IL-12 heterodimer through the IL-12 receptor. However, the sum of their effects would need to restore perforin content, granule polarization, and conjugate formation, since each of these effects is restored by IL-12. These limitations notwithstanding, the current study expands our knowledge of the defective NK cytotoxic activity of patients receiving ART and provides a proof of principle of a possible therapeutic approach.

Our observations may also have clinical implications. The finding that NK cells from HIV-infected patients had defective anticryptococcal activity during viral suppression with ART suggests that damage to immunity from HIV is not adequately corrected by suppressive ART. In contrast to the widespread belief that suppressing viral replication with ART leads to CD4 recovery and restoration of immunity to non-HIV-infected status, our observations add to the body of information that ART restores many aspects of the immune system but not NK cell functions (27, 65). Thus, the defect in NK cells is consistent with a process that is permissive but not sufficient to produce a cryptococcal infection. It is interesting to speculate how this permissive event might be responsible for the high prevalence of subclinical infection associated with cryptococcal antigenemia in HIV-infected patients receiving ART (26).

In conclusion, the current studies show that defective binding, defective polarization of perforin, and defective release of perforin in NK cells from HIV-infected patients are responsible for the aberrant anticryptococcal activity, suggesting that the defects in NK cells from HIV-infected patients are functional and that IL-12 treatment can correct these defects.

## MATERIALS AND METHODS

### Cells and microorganisms.

NK cells from HIV-infected patients and healthy donors were isolated from peripheral blood by negative selection using the RosetteSep human NK cell enrichment cocktail (StemCell, Vancouver, Canada; catalog no. 15065). The purity of CD3^−^ CD16^+^ CD56^+^ NK cells was routinely 94% to 96%. HIV-infected patients receiving ART (*n* = 65) with CD4^+^ counts between 300 and 700 cells/µl and no detectable viral load (<40 copies/ml) and 4 ART-naive patients with CD4^+^ counts of 300 to 600 cells/µl and a viral load of 53,063 ± 68,743 copies were recruited from the Southern Alberta Clinic into this study. The use of human material was approved by the Conjoint Health Research Ethic Board of the University of Calgary, Calgary, AB, Canada. *Cryptococcus neoformans* (ATCC 34873; Manassas, VA) cells were maintained and cultured in Sabouraud dextrose broth and agar (BD Biosciences, Mississauga, Canada) as previously described (22).

### Anticryptococcal activity.

Anticryptococcal activity was assessed by the determination of the number of CFU as previously described (66). Primary NK cells (1 × 10^5^) were pretreated with 100 IU of rh-IL-12 (R & D Systems, MN; catalog no. 219-IL-005) for 20 h or not pretreated and were cocultured with *C. neoformans* (1 × 10^3^) in a 96-well plate (Costar; VWR, PA) at 37°C for 24 h. In some experiments, IL-12-treated NK cells were treated with 20-µM strontium chloride (SrCl_2_) for 24 h (Sigma-Aldrich, Oakville, ON, Canada) as described previously (67) or not treated.

### Determination of stimulated perforin release and intracellular perforin content.

NK cells (1 × 10^5^) were cultured with or without *C. neoformans* for 1 h. The cells were centrifuged at 800 × *g*, and the supernatants were collected. Perforin released into the culture medium was assessed by ELISA (ab46115; Abcam, Toronto, ON, Canada), according to the manufacturer’s instructions. The optical density was measured using a SpectraMax M2e multimode microplate reader (Molecular Devices, Sunnyvale, CA). To determine stimulated perforin release, NK cells were washed with fresh medium prior to stimulation with *C. neoformans*. The supernatants were collected and analyzed for perforin. “Stimulated perforin release” was calculated by subtracting the amount of perforin in supernatants of unstimulated cells from the amount of perforin in supernatant from cells stimulated with *Cryptococcus*. To determine intracellular perforin content, NK cells were fixed and made permeable using Cytofix/Cytoperm (BD Biosciences) according to the manufacturer’s instructions, and perforin in the NK cells was labeled with FITC-conjugated antiperforin antibody (clone δ G9) or isotype-matched control antibody (BD Biosciences). The fluorescence intensity was measured by flow cytometry with a Guava EasyCyte flow cytometer (Guava Technologies, San Francisco, CA). The data were analyzed with the FlowJo software package (Tree Star, Ashland, OR).

### Sample preparation and optical microscopy.

For immunofluorescence labeling, NK cells (1 × 10^6^/ml) were cultured with calcofluor (Sigma-Aldrich)-labeled *C. neoformans* (2 × 10^6^/ml) for 1 h and then fixed with 3.5% paraformaldehyde for 30 min. The cells were washed twice with 1× phosphate-buffered saline (PBS) and made permeable with 1× Perm/Wash (BD Biosciences; catalog no. 51-2091KZ) for 45 min at room temperature. NK cells were then labeled with a 5-µl working concentration of FITC-conjugated antiperforin antibody per 100-µl experimental sample (BD Biosciences; catalog no. 556577; clone δ G9) in fluorescence-activated cell sorting (FACS) buffer (PBS, 1% bovine serum albumin [BSA], 10% fetal bovine serum [FBS], 0.1% NaN_3_ sodium azide) and 0.1 µg/ml of 4′,6-diamidino-2-phenylindole (DAPI) (catalog no. 268298, Calbiochem, Etobicoke, ON, Canada) for nuclear staining and Alexa Fluor-594-conjugated anti-human CD11a antibody (BioLegend, San Diego, CA; catalog no. 30122) and Alexa Fluor-647-conjugated phalloidin (Invitrogen, Burlington, ON, Canada; catalog no. A22284) for F-actin staining. The samples were transferred onto coverslips (Fisher Scientific, Ottawa, ON, Canada; catalog no. 12-542-B), air dried for 30 min, and mounted with ProLong Gold mounting medium (Invitrogen; catalog no. P36930). Samples were prepared on the same day, and images were acquired on the same day with identical microscope settings.

The images were captured with Volocity acquisition software version 6.2.3 (PerkinElmer, Waltham, MA) on a wide-field fluorescence microscope (Olympus, Richmond Hill, ON, Canada), using a PlanoApo 60×/1.40-numerical-aperture (NA) objective. To further analyze the synapse of natural killer cell and *C. neoformans*, high-resolution confocal images in 3D stack (0.2 μm apart) were acquired using the laser scanning module (LSM800) of a Zeiss Elyra microscope (Carl Zeiss, Toronto, ON, Canada) with the aid of a 63×/1.4-NA objective. To prepare a 3D model, stacks of images were first imported in ImageJ (http://rsbweb.nih.gov/ij/docs/faqs.html), using the Bio-Formats importer plugin (68). The stacks of images were then visualized by a 3D viewer, under volume view. A movie was recorded by spinning the 3D model to 360°.

### Image analysis.

Two-dimensional (2D) images were analyzed with Volocity image analysis software version 6.2.3. NK cells that were bound to *C. neoformans* as seen by phase contrast were selected for all conditions. The center of the point of contact was defined as the midpoint of the traced contact interface between an NK cell and *C. neoformans*. The spot function of Volocity was used to identify the perforin-containing granules, and the same threshold of intensity was manually set and applied to both control and test samples. The areas of fluorescence emission that were smaller than 0.05 µm in diameter were excluded, as they were considered not to represent granules. The same threshold settings of fluorescence intensity and size of granules were used in analyzing both the control and test samples on the same day. The distance of perforin-labeled granules to *C. neoformans* was assessed by measuring the distance from the centroid of each granule in an NK cell to the center of the point of contact with *C. neoformans*. The average measurement represents the nonweighted average of the granule–point-of-contact-center distances. The mean of the distances generated per cell was calculated, which represents one data point. The data were exported to GraphPad Prism (GraphPad Software Inc., La Jolla, CA) and graphed. Contrast and brightness of the images were enhanced for visualization purpose only with no distortion, elimination, or obscuring of any structure in the original image, using Volocity image analysis software version 6.2.3. Control and experimental images are presented using identical settings for brightness and contrast.

### Binding and conjugate formation.

Assessment of conjugate formation of NK cells with *C. neoformans* was done as described previously (69). Briefly, NK cells were labeled with PE-Cy5-conjugated anti-CD11a antibody (BD Biosciences), and *C. neoformans* was labeled with 0.1 µM FITC (Sigma; catalog no. 3326-32-7). NK cells were mixed with *C. neoformans* and incubated for 1 h or as specified before the reaction was stopped with 3% formalin at the end of the incubation period. NK cell binding to *C. neoformans* was analyzed by flow cytometry. In some experiments, the PE-Cy5-CD11a-labeled NK cells were cocultured in the presence of polyclonal anti-NKp30 blocking antibody or rabbit IgG (ab27472; Abnova, Taiwan).

### Statistical analyses.

Statistical analyses were performed using GraphPad Prism v6.0. Unless otherwise specified, one-way analysis of variance (ANOVA) followed by Bonferroni comparison tests or unpaired *t* tests (two-tailed) with Welch correction was used to determine differences among conditions. Statistical significance was achieved if *P* was <0.05.

## SUPPLEMENTAL MATERIAL

Figure S1  FITC labeling does not affect viability or binding of *C. neoformans* to NK cells. (A) *C. neoformans* strain B3501 was incubated with or without FITC (7.7 µM final concentration) at 37°C for 5 min, washed three times with PBS, and incubated at 37°C for 24 h, and CFU were assessed. (B) Primary NK cells (2 × 10^6^ cells/ml) were isolated from healthy donors, cocultured with FITC-labeled and unlabeled *C. neoformans* (5 × 10^5^ cells/ml), and imaged at 37°C using a Zeiss Elyra microscope. *Cryptococcus* was deemed in contact with an NK cell if they were less than 1 µm from each other. The percentage of *Cryptococcus* cells in contact with NK was calculated as (number of *Cryptococcus* cells in contact with NK)/(total number of *Cryptococcus* cells in field) × 100%. Twenty different fields containing *Cryptococcus* were chosen at random. The mean from the 20 fields was calculated, and the error bars represent standard errors of the means. Significance was determined by Student’s *t* test. Download Figure S1, TIF file, 3.2 MB

Movie S1  3D model of interaction of an NK cell with *C. neoformans* from a healthy donor. Red, LFA-1; green, perforin; white, differential interference contrast (DIC). Contrast for DIC had been increased artificially to show the outer boundary of *C. neoformans*. The angle of rotation and the orientation of the cell are shown at the corner of the movie by the axes. A 360° rotation at a rate of 3 frames/s is displayed. Download Movie S1, AVI file, 3.7 MB

Movie S2  3D model of interaction of an NK cell with *C. neoformans* from an HIV-infected patient. Red, LFA-1; green, perforin; white, differential interference contrast (DIC). Contrast for DIC had been increased artificially to show the outer boundary of *C. neoformans*. The angle of rotation and the orientation of the cell are shown at the corner of the movie by the axes. A 360° rotation at a rate of 3 frames/s is displayed. Download Movie S2, AVI file, 1.5 MB
