# Supplementary figures and images for: Mechanisms by Which Interleukin-12 Corrects Defective NK Cell Anticryptococcal Activity in HIV-Infected Patients
Source: mBio. 2016 Aug 23;7(4):e00878-16. doi: 10.1128/mBio.00878-16 (PMC4999542; doi:10.1128/mBio.00878-16)

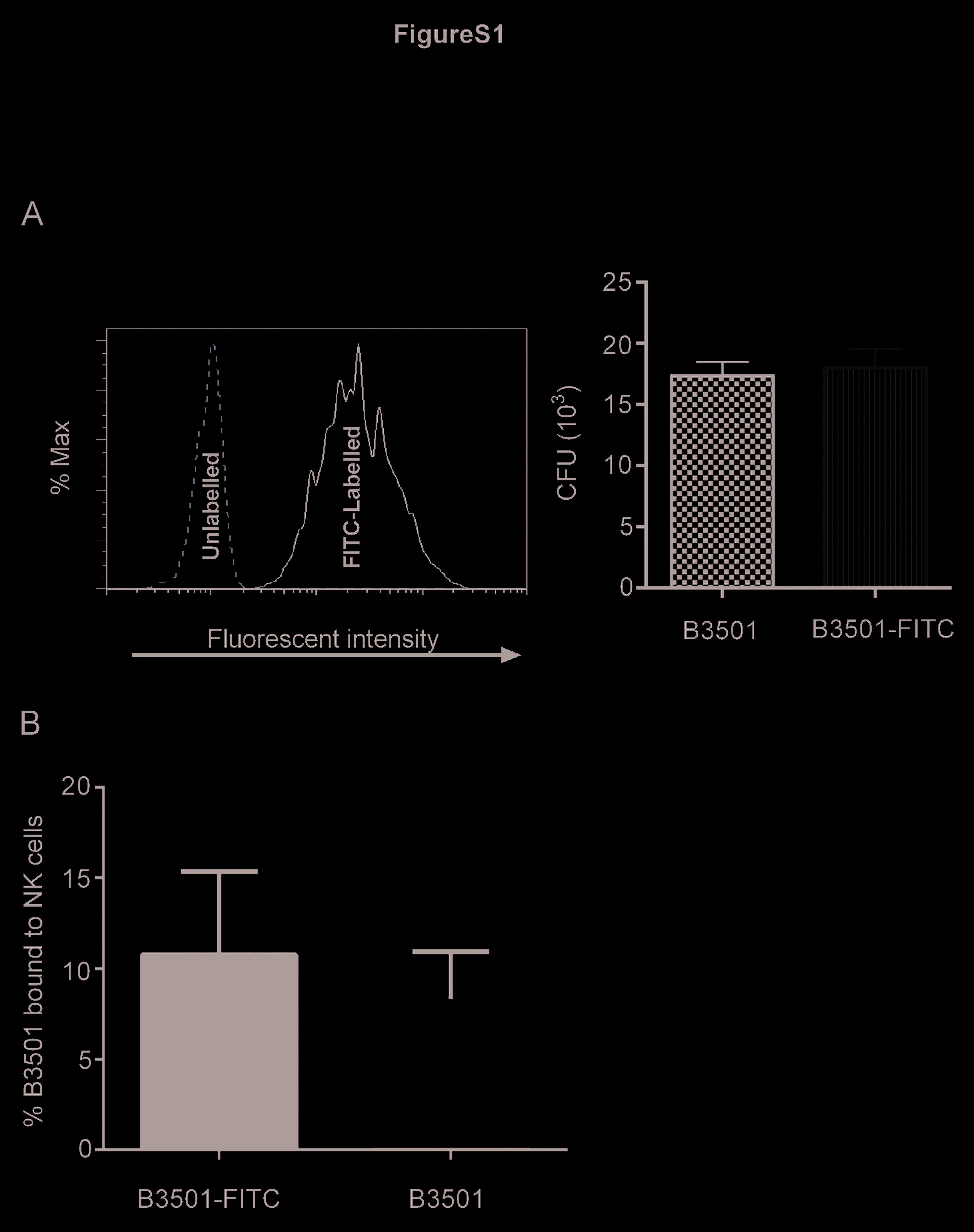

Supplement: Figure S1 — FITC labeling does not affect viability or binding of C. neoformans to NK cells. (A) C. neoformans strain B3501 was incubated with or without FITC (7.7 µM final concentration) at 37°C for 5 min, washed three times with PBS, and incubated at 37°C for 24 h, and CFU were assessed. (B) Primary NK cells (2 × 10^6^ cells/ml) were isolated from healthy donors, cocultured with FITC-labeled and unlabeled C. neoformans (5 × 10^5^ cells/ml), and imaged at 37°C using a Zeiss Elyra microscope. Cryptococcus was deemed in contact with an NK cell if they were less than 1 µm from each other. The percentage of Cryptococcus cells in contact with NK was calculated as (number of Cryptococcus cells in contact with NK)/(total number of Cryptococcus cells in field) × 100%. Twenty different fields containing Cryptococcus were chosen at random. The mean from the 20 fields was calculated, and the error bars represent standard errors of the means. Significance was determined by Student’s t test. Download [file mbo004162937sf1.tif]
